# Case of Lateral Curvature of the Spine Cured by Keeping the Patient in a Recumbent Posture

**Published:** 1822-09

**Authors:** S. D. Broughton

**Affiliations:** Surgeon to the St. George's and St. James's Dispensary, and to His Majesty's Second Regiment of Life-Guards.


ART. III.—*Case of Lateral Curvature of the Spine cured by keeping the Patient in a recumbent Posture.* By S. D. BROUGHTON, Surgeon to the St. George's and St. James's Dispensary, and to His Majesty's Second Regiment of Life-Guards.

THE subject of the following case affords an example of the advantages attendant upon a strict discrimination between the different forms of disease usually known by the too general classification of "spine cases."

When the patient leans on one side and projects the hip of the other, drops one shoulder, and has an evident unevenness of the surface of the chest, is much debilitated and disordered in health, it will usually be found, upon examination, that the spine, instead of forming a straight line down the back, is more or less crooked, and curved somewhat in the form of the letter S. Such a patient is generally of a strumous constitution, and the distortion of the medulla oblongata is attended with impeded digestion, and some interruption to all the healthful functions of life.

The seat of this disorder is most probably in the ligaments which connect the chain of bones together; and, although the distortion of the medulla oblongata creates a disordered state of functions, yet the remote cause of the complaint may be of a constitutional nature, proceeding from bad air or food, hard labour, &c. producing debility of the ligamentous fibres.

Such is the general view which, I believe, may be taken of cases of lateral curvature, in contradistinction to those diseases of the spine which arise from ulceration of the cartilages and carious bone, attended with a projection of the spinous processes of the vertebræ.

Cases of lateral curvature, if neglected, render the patient wholly unfit for the offices of society, the body getting every day more distorted, and its weakness increasing in proportion. The simple plan of treatment required cannot be too quickly adopted, nor too perseveringly submitted to, whilst it totally sets aside all necessity for the too indiscriminate application of issues, caustics, blisters, and instruments. When the weight of the head and trunk is removed from pressing upon the bent column of the vertebræ, and the patient laid straight and quietly on a couch, the spine is disposed to regain its straightness; and, by setting the medulla oblongata free from the unnatural pressure and contortion it suffered, the functions of digestion, &c. become re-established in their healthful state, and the emaciated body acquires flesh and vigour. No farther remedy is called for, unless it be to attend to the regularity of the bowels, to administer some tonic medicine, and to make use of cold affusion or emersion, as means of aiding that on which is to be placed the chief reliance,—the keeping the body horizontally on a couch, without soft beds or a pillow, till nature restores the patient to sufficient strength to enable the spine to sustain the body erect.

Clarissa Honeyset, aged twelve years, was brought to me at the Dispensary in Old Burlington-street, on the 26th of March, 1821. The child was pale, thin, and weak, and had been gradually getting crooked of late, which her parents attributed to weakness of body. Her bowels were apt to be disordered, and she had very little appetite. She usually stood by resting on a table or a chair; the body was thrown on one side, the hip projected on the other, and the chest appeared as if growing out laterally. On examining the spine, it was found to be very much bent on one side in its passage down the back. The mother had been advised to have caustics applied. I recommended her simply to prepare a couch without the usual bedding, and merely to lay some blankets across a board, and without any pillow, so that the head might be as nearly horizontal with the body as possible. Some difficulty was at first encountered in getting the child to lie upon the couch, so prepared, but it was shortly submitted to with more attention. When its bowels were become regular, the tinctura ferri muriate was given, and a column of cold water was poured upon the back every morning, from a tea-kettle held as high as possible.

In a few weeks, I had the pleasure to find the child's health improving: it evidently gained flesh, became ruddy in the face, enjoyed its food, and rested soundly at night. Subsequently the child appeared to be less crooked, and certainly stronger; so that the parents were encouraged to persevere, which they did unremittingly. The child herself also became indifferent to rising, and amused herself with sewing, &c.

In this posture she continued some months, never getting up for more than a few minutes at a time once in twenty-four hours; and, in somewhat less than a year, I found her running about quite strong, straight, and healthy; her parents having found that latterly she seemed to suffer a little from confinement, and, since she had regained her upright position, they relinquished any farther perseverance, taking the precaution to let her lie on the bed occasionally during the day; which I advised the adoption of for some time whilst the girl was growing.

*Great Marlborough-street; July* 1822.

